# The relationship between contact lens ultraviolet light transmittance and myopia progression: a large-scale retrospective cohort study

**DOI:** 10.1093/pcmedi/pbae022

**Published:** 2024-10-11

**Authors:** Hiroyuki Okada, Masao Yoshida, Masaki Takeuchi, Eiichi Okada, Nobuhisa Mizuki

**Affiliations:** Department of Ophthalmology and Visual Science, Yokohama City University Graduate School of Medicine, Yokohama, Kanagawa 236-0004, Japan; Okada Eye Clinic, Yokohama, Kanagawa 234-0054, Japan; Department of Public Health, Kyorin University Faculty of Medicine, Mitaka, Tokyo 181-8611, Japan; Department of Ophthalmology and Visual Science, Yokohama City University Graduate School of Medicine, Yokohama, Kanagawa 236-0004, Japan; Okada Eye Clinic, Yokohama, Kanagawa 234-0054, Japan; Department of Ophthalmology and Visual Science, Yokohama City University Graduate School of Medicine, Yokohama, Kanagawa 236-0004, Japan

**Keywords:** ultraviolet, myopia, progression, cohort study, contact lens

## Abstract

**Background:**

The prevalence of myopia is increasing dramatically around the world, and many studies have suggested the possibility that ultraviolet (UV) light is effective to prevent the onset and progression of myopia. However, UV is a risk factor for diseases that cause refractive errors such as cataract and pterygium. In this study, we evaluated the relationship between UV exposure and myopia progression.

**Methods:**

The dataset consisted of a total of 337 396 eyes of patients in the 12-to-29-year age range, who were prescribed soft contact lenses (SCL) for refractive error at Okada Eye Clinic in Japan between 2002 and 2011. They were tracked over a five-year period and did not change the type of SCL. In this retrospective cohort study based on medical records, we divided patients into two groups, one prescribed SCL with UV protection (UV-SCL), and another prescribed SCL without UV protection (UV + SCL).

**Results:**

Change in refractive power over five years was measured and results compared. It was −0.413 diopter (D) in the UV-SCL group and −0.462 D in the UV + SCL group. Thus, the progression of myopia was slower in the UV-SCL group. The results were also analyzed separately by gender and degree of myopia at the time of initial prescription, which all showed significant differences (*P *< 0.001).

**Conclusion:**

Results suggest that UV exposure may advance myopia. Further research is needed to investigate the underlying mechanisms that could explain this.

## Graphical Abstract

**Graphical Abstract:**
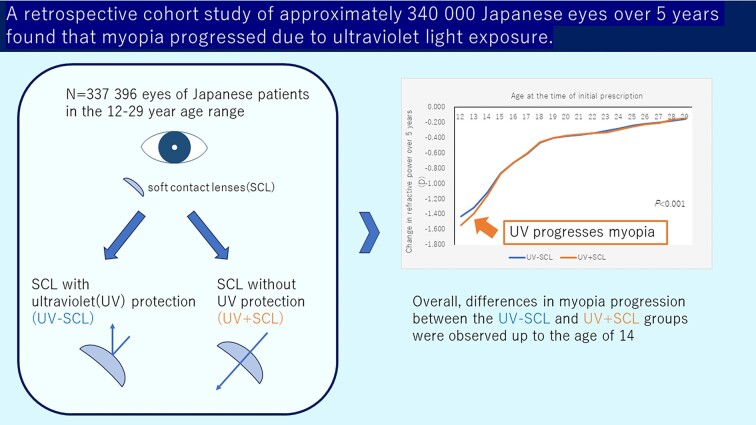

## Introduction

Myopia develops from a mismatch between the optical properties of the tissue in the anterior segment of the eye and the length of the eyeball (axial length) from the cornea to the retina, resulting in distant images being focused in front of the retinal photoreceptors [1]. Besides the functional inconvenience of blurred vision, myopia predisposes people to many blinding diseases in adulthood, such as retinal detachments, glaucoma, and cataract [2]. In particular, high myopia increases the risk of chorioretinal abnormalities such as retinal detachment. Therefore, preventing the onset and progression of myopia is important for public health [3]. Unfortunately, the prevalence of myopia is increasing dramatically worldwide [4–7]. It is estimated that 49.8% of the world's population may be myopic by 2050, and therefore myopia is a major public health concern [8].

Genetic and environmental factors are thought to interact in the onset of myopia, but the mechanism is unknown [7]. However, most myopia develops in childhood, and the prevalence of myopia is rapidly increasing in association with increasing education and urbanization. Some studies suggest that it is likely that the influence of rising education levels associated with economic development outweighs genetic influences [9, 10].

Hyperopia is when distant images are focused behind the retinal photoreceptors, and emmetropia is when the eye has no refractive error [11]. It is widely known that most infants are hyperopic and their eyes become more emmetropic with age [11]. Recently, several studies have suggested that spending time outdoors during childhood correlates with the progression of myopia [12–15]. In a cross-sectional study of 12-year-old children in Sydney, Australia, (*n* = 2367) Rose *et al*. reported that children who spent more total time outdoors had increased hyperopic refraction and a lower prevalence of myopia, whereas children who engaged in high levels of close-up work and low levels of outdoor activity had lower hyperopic refraction [16].

Although the etiology of myopia is still unknown, some have pointed out that close-up work such as reading is a risk factor [17]. On the other hand, there are some studies that suggest that outdoor light is related to the progression of myopia [18–21]. Studies by Ashby *et al*. found that axial length became shorter in chicks exposed to highly luminescent light, and they reported that the release of dopamine due to light stimulation may be involved in the inhibition of the axial length [18, 19]. Torii *et al*. reported that in a chicken myopia model, ultraviolet (UV) light contained in visible light increases the expression of the myopia-suppressing gene *EGR1* which suppresses axial elongation [20]. However, there are some doubts about the application of the chicken myopia model to humans [19–21]. Furthermore, UV is a risk factor for pterygium (the wing-shaped thickening of the conjunctiva and cornea) and juvenile cortical cataract that affect the conjunctiva and crystalline lens. UV has been shown to cause refractive errors. Therefore, it is questionable whether UV light can really be a protective factor for the progression of myopia [22, 23].

Here, a large-scale epidemiological study was conducted on the Japanese population from children to young adults who use soft contact lenses (SCL). The purpose of the study was to clarify the relationship between UV exposure and the progression of myopia by comparing the presence or absence of the UV protection function of SCL.

## Methods

### Study design

The dataset comprised of a total of 2 137 195 eyes of male and female patients who were prescribed SCL for refractive error at Okada Eye Clinic (Yokohama, Japan) between 2002 and 2011. All patients were 12 to 29 years old at the time of first prescription of SCL, as patients aged 12 years or older are most likely to start using SCL, and previous studies have shown the clinical myopia progression may be observed up to the 20s [24].

The refractive power data of the subject group in this study was determined by visual acuity measured using an auto-ref keratometer (KR-3000; TOPCON, Tokyo, Japan) and, if necessary, a skiascope (RX-3ASP; NEITZ, Tokyo, Japan).

SCL was prescribed based on subjective and objective test results. The eyes included in the present study were divided, according to different types of SCL prescribed, into a UV-SCL group (SCL with UV protection; 1 208 274 eyes) and a UV + SCL group (SCL without UV protection; 928 921 eyes). Among the total dataset of 2 137 195 eyes, 337 396 eyes (including 170 151 eyes prescribed UV-SCL and 167 245 eyes prescribed UV + SCL) could be tracked for five years, and the patient had not changed the presence or absence of UV protection function or the type of SCL. Patients with corneal diseases that affected refractive values, patients who had undergone ophthalmic surgery in the past or at present, and patients with intellectual disabilities for whom the test was unreliable, were excluded.

**Figure 1.**
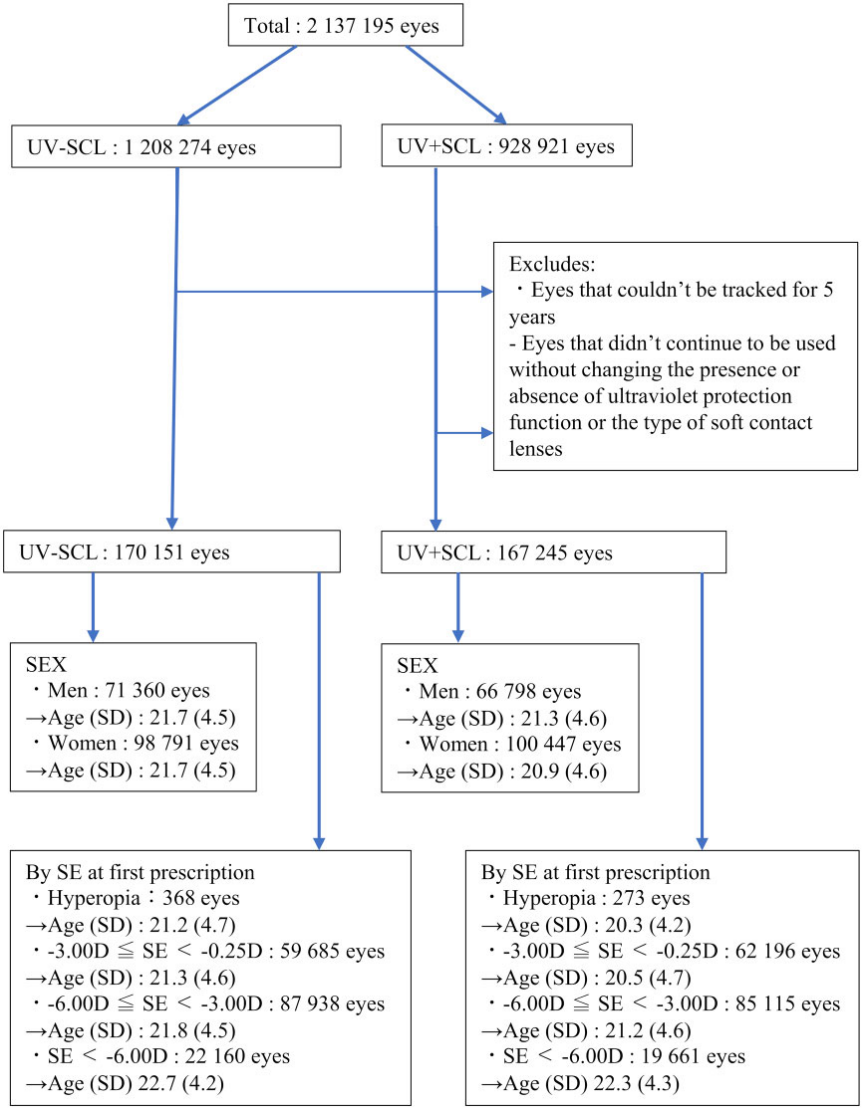
Data flow chart of the study. Total, the eyes of male and female patients aged 12 to 29 years at the time of first prescription which were prescribed soft contact lenses; UV-SCL, the group prescribed soft contact lenses with ultraviolet protection; UV + SCL, the group prescribed soft contact lenses without ultraviolet protection; Age, average age; SD, standard deviation; SE, spherical equivalent power; D, diopters.

This study was a retrospective cohort study based on medical records, and as there was no additional survey of subjects, it was conducted with an opt-out option (Fig. 1 and Supplementary Tables S1–S2 and S5–S6).

In this study, UV was defined as the combined wavelengths of UVA (wavelength 320–400 nm) and UVB (wavelength 280–320 nm). The absorption rate of the UV-SCL used by patients in this study is listed in Supplementary Table S5.

### Statistical analysis

The refractive changes in spherical equivalent power (SE) over a five-year period were determined between two groups by gender and three groups by myopia at the time of initial prescription: high myopia [SE←6.00 diopter (D)], moderate myopia (−6.00 D **≤ **SE←3.00 D), and low myopia (−3.00 D **≤ **SE←0.25 D). The Paired *t*-Test was used to analyze them. Furthermore, hyperopia was excluded from the analysis.

The difference between the UV-SCL group and the UV + SCL group of five-year mean was analyzed. SE change in each category was classified by sex, baseline age, and/or baseline SE level using analysis of covariance (ANCOVA). The difference by sex was analyzed using *P* value for the difference between those groups of five-year mean, SE change adjusted by analysis of covariance (ANCOVA) with baseline age and baseline SE as independent variables. Similarly, when the difference was analyzed by baseline SE, *P* value was calculated for the difference between those groups of five-year mean SE change using ANCOVA with sex and baseline age as independent variables.

All analysis was performed using the SAS statistical software package Version 9.4 (SAS Institute Inc., Cary, NC, USA).

## Results

We studied a total of 337 396 eyes (Fig. 1, Supplementary Tables S1–S2) and compared the amount of change and standard deviation (SD) in refractive power over a five-year period (Table 1, Supplementary Tables S3–S4).

**Table 1.** Change in refractive power over a 5-year period by soft contact lenses.

			SE at baseline :	5-year SE Change	95%CI (D)		*P*
			mean (SD) (D)	mean (SD) (D)			
Sex	Men	UV-SCL	−4.124 (1.948)	−0.431 (0.002)	−0.435	−0.426	<0.001
		UV + SCL	−4.027 (1.860)	−0.467 (0.003)	−0.472	−0.462	
	Women	UV−SCL	−4.020 (1.920)	−0.401 (0.002)	−0.405	−0.397	<0.001
		UV + SCL	−3.870 (1.847)	−0.458 (0.002)	−0.462	−0.454	
SE at first prescription	SE < −6.00D	UV−SCL	−7.603 (1.178)	−0.281 (0.005)	−0.290	−0.272	<0.001
		UV + SCL	−7.412 (0.947)	−0.323 (0.005)	−0.333	−0.314	
	−6.00D ≦ SE < −3.00D	UV−SCL	−4.464 (0.847)	−0.402 (0.002)	−0.406	−0.398	<0.001
		UV + SCL	−4.431 (0.848)	−0.435 (0.002)	−0.439	−0.431	
	−3.00D ≦ SE < −0.25D	UV−SCL	−2.204 (0.614)	−0.479 (0.003)	−0.484	−0.474	<0.001
		UV + SCL	−2.186 (0.624)	−0.541 (0.003)	−0.546	−0.535	
	Total	UV−SCL	−4.063 (1.932)	−0.413 (0.001)	−0.416	−0.410	<0.001
		UV + SCL	−3.933 (1.854)	−0.462 (0.002)	−0.465	−0.458	

*P* value for difference between groups of 5-year mean SE (Spherical equivalent power) change adjusted by analysis of covariance (ANCOVA) with sex, baseline age, and/or baseline SE as an independent variable. SD, Standard deviation; D, Diopters; 95%CI, 95% confidence interval; UV-SCL, A group prescribed soft contact lenses with ultraviolet protection; UV + SCL, A group prescribed soft contact lenses without ultraviolet protection.

Comparing the mean age by gender, men were 0.4 years younger, and women were 0.8 years younger in the UV + SCL group than in the UV-SCL group. On the other hand, when comparing the average age by degree of myopia at the time of first prescription, the UV + SCL groups were 0.4 years younger for high myopia, 0.6 years younger for moderate myopia, and 0.8 years younger for low myopia than the UV-SCL groups (Fig. 1).

SE (mean ± SD) at baseline was −4.063 ± 1.932 D overall in the UV-SCL group. By gender, it was −4.124 ± 1.948 D for men and −4.020 ± 1.920 D for women in the UV-SCL group. In addition, it was −3.933 ± 1.854 D overall in the UV + SCL group; when broken down by gender, it was −4.027 ± 1.860 D for men and −3.870 ± 1.847 D for women (Table 1).

**Figure 2.**
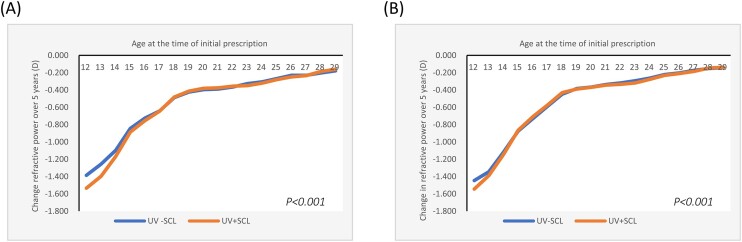
Change in refractive power over a five-year period by soft contact lenses by gender. (A) Men. (B) Women. D, diopters; UV-SCL, the group prescribed soft contact lenses with ultraviolet protection; UV + SCL, the group prescribed soft contact lenses without ultraviolet protection.

**Figure 3.**
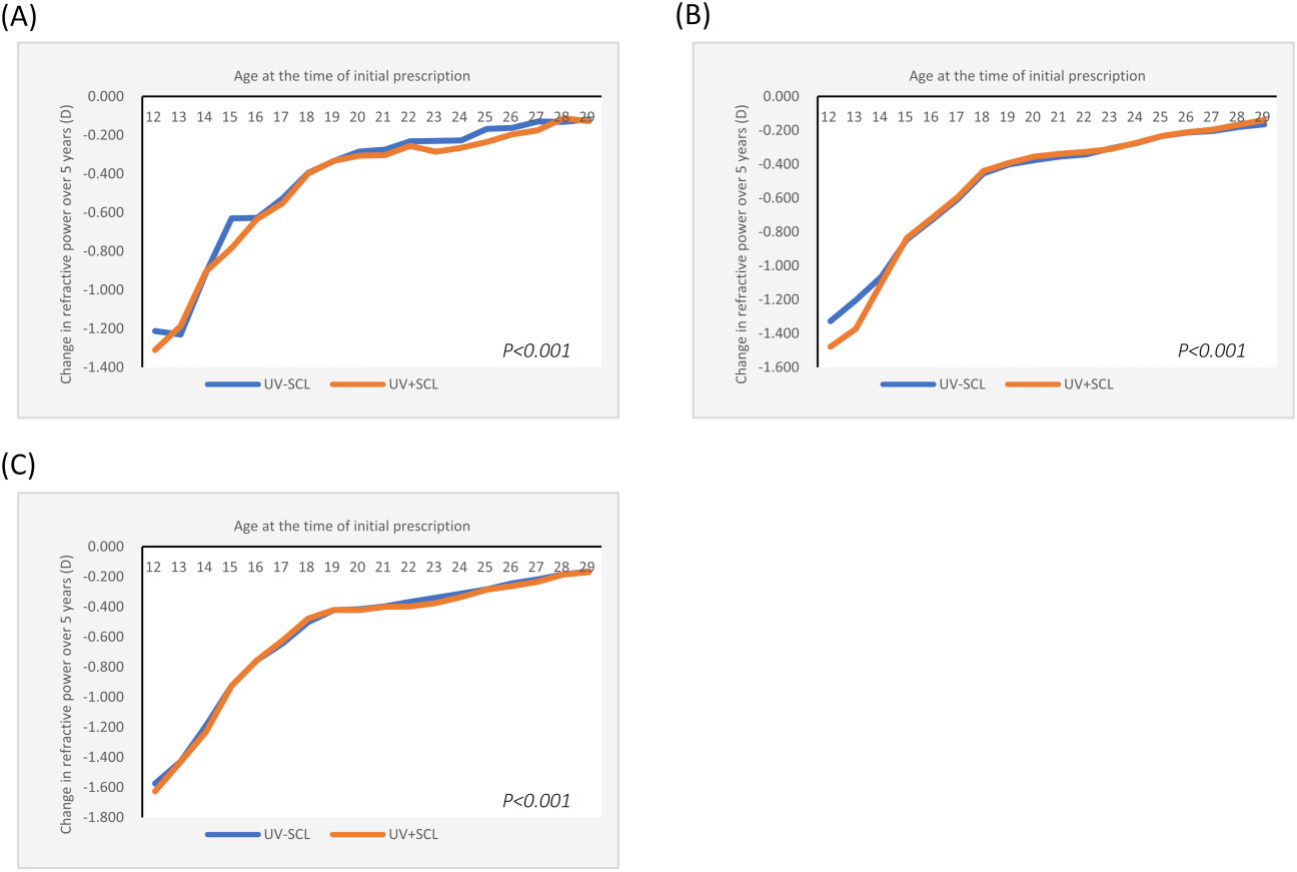
Change in refractive power over a five-year period by soft contact lenses by degree of myopia at the time of initial prescription. (A) High myopia. (B) Moderate myopia. (C) Low myopia. D, diopters; UV-SCL, the group prescribed soft contact lenses with ultraviolet protection; UV + SCL, the group prescribed soft contact lenses without ultraviolet protection.

**Figure 4.**
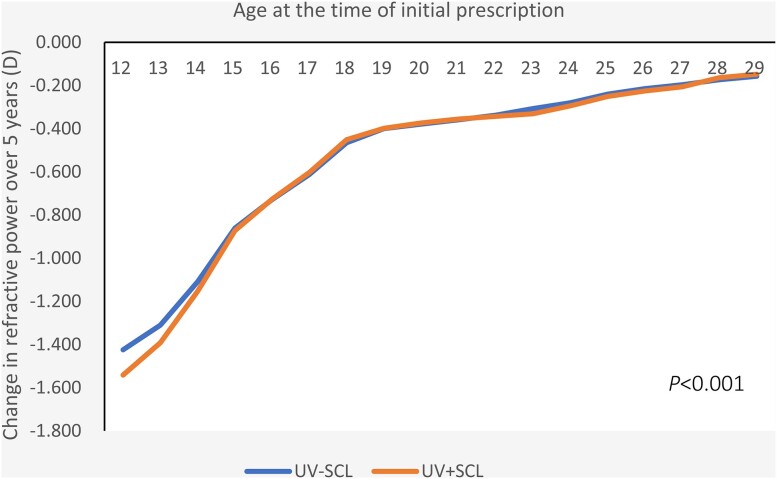
Change in refractive power over a 5-years period (Total). D, diopters; UV-SCL, the group prescribed soft contact lenses with ultraviolet protection; UV + SCL, the group prescribed soft contact lenses without ultraviolet protection.

The overall refractive change in SE over five years was −0.413D (95% CI= −0.416, −0.410) for the UV-SCL group and −0.462 D (95% CI= −0.465, −0.458) for the UV + SCL group. By gender, it was −0.431 D (95% CI= −0.435, −0.426)/−0.467 D (95% CI= −0.472, −0.462) for men, and −0.401 D (95% CI= −0.405, −0.397)/−0.458 D (95% CI= −0.462, −0.454) for women. By myopia power at the time of initial prescription, −0.281 D (95% CI= −0.290, −0.272)/−0.323 D (95% CI= −0.333, −0.314) for high myopia, −0.402 D (95% CI= −0.406, −0.398)/−0.435 D (95% CI= −0.439, −0.431) for moderate myopia, and −0.479 D (95% CI= −0.484, −0.474)/−0.541 D (95% CI= −0.546, −0.535) for low myopia. In either case, myopia progression was larger in the UV + SCL group than in the UV-SCL group (*P *< 0.001 for all) (Table 1, Figs. 2–4 and Supplementary Tables S3–S4).

By gender, differences in myopia progression between the UV-SCL and UV + SCL groups were observed up to 16 years of age for men and 13 years of age for women (Fig. 2, Supplementary Tables S3–S4).

On the other hand, when looking at the degree of myopia at the time of the first prescription, a difference in the progression of myopia up to the age of 13 was only observed with moderate myopia (Fig. 3, Supplementary Tables S3–S4).

Overall, differences in myopia progression between the UV-SCL and UV + SCL groups were observed up to the age of 14 (Fig. 4, Supplementary Tables S3–S4).

## Discussion

Up to 80% of a person's lifetime UV exposure is reached before the age of 18, so it is recommended that UV-blocking spectacles or contact lenses are worn from a young age [23]. In the Yokohama study that examined refractive changes over a five-year period in Japanese eyes (*n* = 593 273), the largest change was observed in both sexes at the age of 8 years. However, change in myopia decreased with age, and clinical myopia stopped at the age of 27 years in males and 26 years in females [24]. Furthermore, it has been suggested that UV-blocking contact lenses not only reduce the oxidative damage caused by UV to ocular tissues, but also may delay presbyopia when worn for a long time [25].

The results of this study showed that myopia progressed in the UV-exposed group. However, there are those who suggest that UV exposure can inhibit the progression of myopia, in particular, studies on violet light (VL: wavelength 360–400 nm), short-wavelength visible violet light that is a component of sunlight [20, 26–30]. In a cohort study of Japanese children aged 6 to 12 years and 10 to 15 years (*n* = 113, 310), Torii *et al*. reported that elongation of axial length was shorter in children using VL transmitting eyeglasses and contact lenses than in children using VL cutting eyeglasses, thus proposing that VL suppresses myopia. Contrary to such proposal [20, 26], our research suggested that myopia progresses with UV exposure. However, since this study only compared the presence and absence of UV protection, effects of viewing distance and environmental factors that occur when comparing indoors and outdoors were small. In addition, since the dataset was large, other biases were also considerably small. It is therefore argued that the current study had a high degree of reliability.

The reason for doubts about the applicability of the chicken myopia model to humans is that humans have much less sensitivity in the near-UV region of the spectrum than chicks [21]. Torii *et al*. reported that VL may suppress myopia and elongation of axial length in children, but unlike humans, chicks are sensitive to near-UV light and transmit light up to at least 360 nm. The human crystalline lens transmits only 1% of light below 400 nm. It remains unclear how UV illumination affects myopia in humans [20, 21, 26].

Studies examining myopia and hyperopia under monochromatic light of different wavelengths have been conducted in various animals. Under short wavelength violet light (wavelength 380–420 nm) and blue light (wavelength 465 nm), the progression of myopia was reduced in chickens, mice, guinea pigs, and African cichlid fish [20, 21, 28–35]. On the other hand, two mammalian species, rhesus macaques [36] and shrews [37, 38], showed a reduction in the progression of myopia under long-wavelength red light (wavelength 630 nm), opposite to the other studies. However, some have pointed out that long-wavelength red light during infancy may be a risk factor for developing myopia in a small proportion of rhesus macaques who are sensitive to rhesus L-cone stimulation [39].

There are various opinions regarding the mechanism by which monochromatic light affects the development of nearsightedness and farsightedness. For example, it has been suggested that cone signaling may play a role in hyperopic responses to short wavelengths in mutant mice with dysfunctional cones [28]. On the other hand, a study using guinea pigs has indicated that retinal cones/opsins are related to refraction [29]. However, there is no established mechanism yet.

In addition, since the wavelengths of light to which each test animal is sensitive differs, it is difficult to determine which category humans fall under. Since rhesus macaques also have individual differences in wavelength sensitivity, it is possible that there may be racial differences in humans as well [39].

In fact, the prevalence of myopia is increasing, especially in urban areas in East Asia [7]. The idea that populations of East Asian origin have an inherently higher prevalence of myopia is not supported by the very low prevalence reported for those living in rural areas, and by the high prevalence of myopia reported among Indians living in Singapore [9]. Environmental change may be a major factor in increasing the prevalence of myopia around the world [9]. Although it is believed that individuals differ in their susceptibility to environmental risk factors, evidence to support this idea is lacking [9].

It is hoped that this will become clearer in the future through verification of many studies including this one. It may be important to reconsider the relationship between time spent outdoors during childhood and the progression of myopia [12–15]. In a meta-analysis of 25 papers (*n* = 34 420) reporting on outdoor activity time, an increase in outdoor activity time reduced the risk of developing myopia but was not effective in slowing the progression of myopia for myopic eyes [40].

Furthermore, in a study of students in an area of Central Finland (*n *= 4305), children who spent a long time indoors had a close reading distance and were at higher risk for myopia [41]. A study of Spanish children aged 5 to 7 years (*n *= 7497) found that myopic children were exposed to more screentime and spent less time outdoors [42].

However, in a study which considered the prevalence of myopia among people of the same age and ethnicity, the prevalence rate was 3.3% for Chinese Australians aged 6 to 7 years, compared with 29.1% for Chinese Singaporeans of the same age. Although the prevalence of myopia was significantly lower in the Chinese Australians, Australian children spent more time outdoors and more time engaged in close-up activity. Rose *et al*. hypothesize that the pressure of early education in Singapore may be the cause [43].

While the relationship between time spent indoors or outdoors and myopia may be related to viewing distance, potential confounders such as education level, occupation, and socio-economic status may be associated with both myopia and time spent outdoors [43, 44]. Nowadays, it is possible to use smart watches to collect data such as luminance, UV levels, number of steps, and indoor and outdoor location. Efforts to collect more data using such devices in the future may help to uncover the cause [44].

One of the limitations of this study is that it was desirable to perform cycloplegia to accurately measure refractive value because children have strong eye accommodation capabilities. However, this was not done in the present study based on SCL prescription data. On the other hand, it has been reported that the difference in refractive values ​​with and without cycloplegia becomes smaller with age, and there is no difference in those aged 12 years or older [45]. Therefore, in the present study, which targeted children aged 12 years or older, it is considered that the impact of not performing cycloplegia on refractive value ​​is small.

Many studies about refractive error often focus on axial length. However, since this study was based on prescription data for SCL, it was difficult to secure sufficient data on the axial length of the target group. Since the cornea and crystalline lens are also related to refraction, refractive value needs to be evaluated comprehensively.

The data from this study should be deemed credible, since the SCL prescription was determined by both subjective and objective test results, and patients consented to purchasing the product. Furthermore, this study did not only use a single brand of SCL, since different types of SCL have different UV absorption rates, it is difficult to consider specific wavelengths and there may be some bias due to SCL type (Supplementary Table S5). However, although manufacturers tend to differ depending on the presence or absence of UV functions, the price range remains similar. Additionally, environmental, lifestyle, and genetic factors that pose a risk for refractive changes were not considered.

A further drawback of the study may be that because *n* was very large, the results may have been overestimated. Since SCL are recommended for use at ages 12 and older, sufficient *n* was not available for data at age 8, when the change in myopia is greatest [24].

It should be noted that the difference in average age between UV-SCL and UV + SCL is slightly larger amongst women. In young people, differences in age and gender may have a strong influence on the progression of myopia. In this study, we performed comparisons by age and gender to minimize bias.

Outdoor activities and close-up work affect the progression of myopia, so it is desirable to take these into consideration, but this study did not examine individual activities. However, because the sample size in this study is very large, it is thought that the influence of bias due to differences in individual activities will be reduced.

The proportion of women choosing UV + SCL decreases with age, and it cannot be denied that women use other UV protection as they get older, the present study did not account for this, and further investigation is considered necessary (Fig. 1 and Supplementary Table S1).

The results showed that UV + SCL had lower baseline refractive power both overall and by gender. However, this result is only a trend in the refractive power of each group and is not believed to have had any effect on the result that myopia progresses more with UV + SCL than with UV-SCL.

## Conclusion

Taken together, results of the present study with a large cohort suggest that UV exposure may be a risk factor for the progression of myopia.

## Supplementary Material

pbae022_Supplemental_Files

## Data Availability

Data used in this study was obtained from the refractive power of soft contact lenses database, which is managed by Okada Eye Clinic. The society allows members to use the data for scientific research but does not allow anyone to share original data publicly.
